# *Besnoitia besnoiti* lytic cycle in vitro and differences in invasion and intracellular proliferation among isolates

**DOI:** 10.1186/s13071-016-1405-9

**Published:** 2016-02-29

**Authors:** Caroline F. Frey, Javier Regidor-Cerrillo, Nelson Marreros, Paula García-Lunar, Daniel Gutiérrez-Expósito, Gereon Schares, Jitender P. Dubey, Arcangelo Gentile, Philippe Jacquiet, Varda Shkap, Helder Cortes, Luis M. Ortega-Mora, Gema Álvarez-García

**Affiliations:** SALUVET, Animal Health Department, Faculty of Veterinary Sciences, Complutense University of Madrid, Ciudad Universitaria s/n, 28040 Madrid, Spain; Institute of Parasitology, Vetsuisse Faculty, University of Bern, Länggass-Strasse 122, 3012 Bern, Switzerland; Centre for Fish and Wildlife Health, Vetsuisse Faculty, University of Bern, Länggass-Strasse 122, 3012 Bern, Switzerland; Friedrich-Loeffler-Institut, Federal Research Institute for Animal Health, Institute of Epidemiology, Greifswald, Insel Riems Germany; United States Department of Agriculture, Agricultural Research Service, Beltsville Agricultural Research Center, Animal Parasitic Diseases Laboratory, Beltsville, MD 20705-2350 USA; Department of Veterinary Medical Sciences, University of Bologna, Ozzano dell’Emilia, Italy; Institut National Polytechnique (INP), Ecole Nationale Vétérinaire de Toulouse (ENVT), UMR1225, IHAP, Equipe « Besnoitiose et vecteurs », Université de Toulouse, Toulouse, France; Division of Parasitology, Kimron Veterinary Institute, P.O. Box 12, Bet Dagan, 50250 Israel; Laboratório de Parasitologia Victor Caeiro, Núcleo da Mitra, ICAAM, Universidade de Évora, Apartado 94, 7000-554 Évora, Portugal

**Keywords:** *Besnoitia besnoiti*, *Besnoitia tarandi*, Lytic cycle, In vitro model, Isolates, Invasion, Proliferation

## Abstract

**Background:**

Bovine besnoitiosis, caused by the protozoan *Besnoitia besnoiti*, reduces productivity and fertility of affected herds. Besnoitiosis continues to expand in Europe and no effective control tools are currently available. Experimental models are urgently needed. Herein, we describe for the first time the kinetics of standardised in vitro models for the *B. besnoiti* lytic cycle. This will aid to study the pathogenesis of the disease, in the screening for vaccine targets and drugs potentially useful for the treatment of besnoitiosis.

**Methods:**

We compared invasion and proliferation of one *B. tarandi* (from Finland) and seven *B. besnoiti* isolates (Bb-Spain1, Bb-Spain2, Bb-Israel, Bb-Evora03, Bb-Ger1, Bb-France, Bb-Italy2) in MARC-145 cell culture. Host cell invasion was studied at 4, 6, 8 and 24 h post infection (hpi), and proliferation characteristics were compared at 24, 48, 72, 96, 120, and 144 hpi.

**Results:**

In *Besnoitia* spp., the key parameters that determine the sequential adhesion-invasion, proliferation and egress steps are clearly distinct from those in the related apicomplexans *Toxoplasma gondii* and *Neospora caninum. Besnoitia* spp. host cell invasion is a rather slow process, since only 50 % of parasites were found intracellular after 3–6 h of exposure to host cells, and invasion still took place after 24 h. Invasion efficacy was significantly higher for Bb-France, Bb-Evora03 and Bb-Israel. In addition, the time span for endodyogeny to take place was as long as 18–35 h. Bb-Israel and *B. tarandi* isolates were most prolific, as determined by the tachyzoite yield at 72 hpi. The total tachyzoite yield could not be predicted neither by invasion-related parameters (velocity and half time invasion) nor by proliferation parameters (lag phase and doubling time (dT)). The lytic cycle of *Besnoitia* was asynchronous as evidenced by the presence of three different plaque-forming tachyzoite categories (lysis plaques, large and small parasitophorous vacuoles).

**Conclusions:**

This study provides first insights into the lytic cycle of *B. besnoiti* isolates and a standardised in vitro model that allows screening of drug candidates for the treatment of besnoitiosis.

**Electronic supplementary material:**

The online version of this article (doi:10.1186/s13071-016-1405-9) contains supplementary material, which is available to authorized users.

## Background

*Besnoitia besnoiti*, a tissue cyst-forming apicomplexan protozoan parasite, is the etiological agent of bovine besnoitiosis. Bovine besnoitiosis is a chronic and debilitating disease of economic importance that affects fertility and can lead to reduced production efficacy (reviewed in [1]). *B. besnoiti* has gained the attention of Veterinary Health officials in Europe since bovine besnoitiosis has been identified in previously unaffected regions of this continent during the last years [2]. From the initially recognised foci in Alentejo in Portugal and the Pyrenees, the disease has spread almost all over Spain and France, and has now been observed in Italy, Germany, Switzerland, and most recently in Hungary, Croatia, and Belgium [3–5]. Currently, there are no effective drugs, and no vaccine is licensed in Europe. Closely related to *B. besnoiti* are *B. tarandi* that mainly infects reindeer and other wild ungulates [6], *B. caprae* that mainly affects goats [7], and *B. bennetti*, a parasite infectious for equids [8].

The life-cycles of these species mentioned above have not yet been elucidated to date. Cats are definitive hosts for several other *Besnoitia* species of small mammals [9]. A heteroxenous life-cycle is suspected for *B. besnoiti*, but the definitive host still remains elusive [10, 11]. Cattle represent an important intermediate host for the parasite [12], and harbour tachyzoites and the cyst-forming bradyzoites. Upon infection, tachyzoites undergo proliferation in endothelial cells and fibroblasts [12, 13]. During this acute phase of the disease, animals develop fever, nasal and ocular discharge, photophobia, edema, and lameness [12–16]. The chronic form of besnoitiosis is characterised by the presence of thick-walled tissue cysts containing bradyzoites. Predilection sites for the cysts are the mucosae of the upper respiratory tract, of the lower genital tract, the scleral conjunctiva of the eyes, and the dermis and tendons of the lower limbs [12, 15–18]. In endemic situations, most animals remain subclinically infected with only about 10 % of animals developing the characteristic disease [19, 20]. The reasons why only a small percentage of animals develop disease are not yet understood. There are parasite- and host-related factors that may influence the outcome of the infection (reviewed in [21]). Therefore, standardised in vitro and in vivo models for *B. besnoiti* are urgently needed to study the pathogenesis of the infection and to assess novel drug or vaccine candidates. Several in vitro models have been employed to isolate and propagate *Besnoitia* parasites, even to test a few drug candidates [22, 23]. However, the lytic cycle has not been described in detail yet, and time points of adhesion-invasion, proliferation and egress may differ from the ones reported in other apicomplexans, or even between isolates. This knowledge is essential to enable comparable studies. It is well known that in other closely related tissue cyst-forming apicomplexan parasites, such as *Toxoplasma gondii* and *Neospora caninum*, parasite invasion and proliferation are significantly influenced by parasite strain, host cell line, and activation state, and these phenotypic traits have been associated with virulence [24–26]. Intra-specific variability has been addressed in molecular and virulence studies in *T. gondii* and *N. caninum* [24, 26–33]. For *T. gondii* in Europe and North America, a population structure was demonstrated that is dominated by three major clonal lineages (Types I, II, and III) displaying different behaviour in vitro, contrasting virulence in the mouse model, and distinct genetic hallmarks (reviewed in [24]). In other parts of the world, especially South America and Asia, highly variable atypical strains of *T. gondii* circulate [30, 34], some associated with high virulence for humans [35]. For *N. caninum*, genetic differences among various isolates could be demonstrated [31–33], and interestingly, the in vivo virulence in mice and in cattle could be linked to distinct in vitro behavior in a standardised cell culture assay [25, 36]. So far, similar approaches have not been carried out in *B. besnoiti*. One study analysing four *B. besnoiti* isolates of different countries found genetic differences in at least four microsatellites [37], whereas more recently Gutiérrez-Exposito et al. reported that out of 11 identical *B. besnoiti* isolates one differed at one microsatellite [38]. However, it is unknown whether different *B. besnoiti* isolates differ in their in vitro characteristics and finally also in their virulence.

We here report on the first detailed study on the kinetics of the lytic cycle of different *Besnoitia* spp. isolates in MARC-145 cell culture. The characteristics of host cell invasion and proliferation of one *B. tarandi* and seven *B. besnoiti* isolates from six countries and two continents were studied and compared. Knowledge on the lytic cycle of *Besnoitia* spp. is essential to understand infection kinetics of this parasite. Furthermore, this study demonstrates proof-of-concept for a standardised in vitro model that now allows to compare isolates and to perform meaningful screening of drug candidates.

## Methods

### *Besnoitia* isolates and cell culture

*B. besnoiti* isolates were obtained from Spain (Bb*-*Spain1, Bb-Spain2), Germany (Bb-Ger1), Portugal (Bb-Evora03), Israel (Bb*-*Israel), and from France (Bb*-*France). From an affected cow in Italy we obtained tissue samples and isolated a new Italian isolate, *B. besnoiti* Italy2 (Bb-Italy2) (Table 1). All isolates were tested for the presence of *Mycoplasma* spp. infection by PCR (Venor™GeM Mycoplasma Detection Kit; Minerva Biolabs, Berlin, Germany) and they were only used if *Mycoplasma*-free, as *Mycoplasma* spp. infections affect the host cells.

**Table 1:** *Besnoitia* spp. isolates used in in vitro assays

Isolate	Isolated from^a^	Country	Location	Year of isolation	Reference	Passage numbers (after HFF) used in experiments
Bb-Spain1	Brown Swiss cow	Spain	Guadalajara	2005	[39]	6 to 31
Bb-Spain2	Cow	Spain	Huesca	2005	unpublished	6 to 18
Bb-Italy2	Limousin Cow	Italy	Emilia Romagna Appenines	2012	this study	3 to 11
Bb-Evora03^b^	Sallers cow	Portugal	Evora	2003	[63]	2 to 14
Bb-Israel	Cow	Israel	Golan Heights	1986	[52]	3 to 18
Bb-Ger1^b^	Charolais bull	Germany	Bavaria	2008	[41]	2 to 15
Bb-France	Cow	France	French Pyrenees		[55]	3 to 13
*B. tarandi*	Reindeer	Finland	ND	2004	[6]	6 to 11

^a^All isolates originate from animals with visible tissue cysts

^b^Isolates with a previous passage through interferon-gamma gene knockout (KO) mice

ND: No data available

All isolates were maintained in monolayer cultures of a monkey kidney cell line (MARC-145 cells) employing a parasite-host cell ratio of 2–5:1, in Dulbecco’s modified Eagle medium (DMEM) supplemented with 10 % fetal bovine serum (FBS), 15 mM HEPES (pH 7.2), 2 mM glutamine, penicillin (100 U/ml), streptomycin (100 μg/ml) in 5 % CO_2_/37 °C. FBS was previously checked for absence of anti-*Besnoitia*, anti-*Toxoplasma*, and anti-*Neospora* IgG by IFAT [39].

Tachyzoites were harvested 3 days post-infection (p.i.), when most tachyzoites were still intracellular, and parasites were liberated from their host cells by passing them repeatedly through a G25 needle, followed by purification through disposable PD-10 columns (Sephadex G-25; GE-Healthcare, Buckinghamshire, UK) as described earlier for *Neospora* [25]. Tachyzoite viability was confirmed by trypan blue exclusion and they were counted in a Neubauer chamber. Purified tachyzoites were employed in invasion and proliferation assays.

In order to minimise artefacts due to prolonged maintenance in MARC- 145 cells, all isolates were passaged 4 times through Human Foreskin Fibroblast (HFF) cells, a cell-line they had not been cultured in previously, before being used in the experiments. Afterwards, they were maintained in MARC-145 and used in low passages (Table 1) for the invasion and proliferation experiments.

### Invasion assay

Invasion assays were carried out using confluent MARC-145 cell monolayers grown in 24 well plates (10^5^ cells/well in DMEM supplemented with 10 % FCS). For infection, the medium supernatant was discarded and monolayers were exposed to tachyzoites suspended in 1 ml medium. In a preliminary experiment, infections were carried out using 100, 200, 500, 1000 and 2000 tachyzoites per well in order to establish a reliable and optimised protocol. Finally, 1000 tachyzoites suspended in DMEM supplemented with 5 % FCS were used for infection, resulting in a parasite: host cell ratio of 1:100. All time points were assessed in triplicates.

Infected cultures were further maintained at 37 °C, 5 % CO_2_. Initially, invasion was measured after 1 h post infection (hpi), 2, 4, 6, 8, and 24 hpi. For the experimental series, only time points 4, 6, 8, and 24 hpi were retained, because after 1 or 2 hpi, almost no invasion events could be detected. At those time points, the cell monolayer was washed three times with DMEM 5 % FCS to remove non-invaded tachyzoites. Then, 1 ml DMEM 5 % FCS per well was added and the plates were incubated for a total of 72 h. For each isolate, three infected wells were left undisturbed without washing for the entire 72 h. Invasion was measured by counting the number of plaques per well by IFAT assuming that single tachyzoites caused these plaques (see 2.4.). Invasion assays were repeated three times in independent experiments for each isolate. *Besnoitia* strain Bb-Spain1 was included as an internal control of reproducibility in each series.

### Proliferation assay

For the proliferation assay, 24 well plates with confluent MARC-145 cells (10^5^ cells/well) in DMEM 10 % FCS were used. Monolayers were infected with 10^6^ purified tachyzoites/well suspended in 1 ml DMEM 5 % FCS. After 4 h, the wells were washed three times with DMEM 5 % FCS and infected monolayers were further cultured at 37 °C, 5 % CO_2_. At time points 24, 48, 72, 96, 120, and 144 hpi, three wells per *Besnoitia*-isolate were harvested as follows: the supernatants were discarded and the cells were incubated in 100 μl PBS, 100 μl lysis buffer, 10 μl proteinase K (>600 mAU/ml) per well (Qiagen, Hilden, Germany) for 5 min. The lysates were transferred to Eppendorf tubes and stored at −20 °C until further quantitative real-time PCR analysis (see section 2.5). In preliminary experiments performed with Bb-Spain1, no proliferation was observed earlier than 24 hpi, and after 144 hpi all MARC-145 cells were lysed. Proliferation assays were carried out in triplicates, and were repeated in three independent experiments for each isolate. Bb*-*Spain1 was included as an internal control of reproducibility in each experimental series.

### Assessment of invasion assays by immunofluorescence (IFAT)

**Fig. 1:**
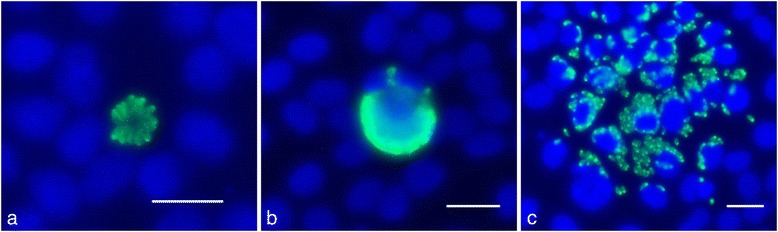
Immunofluorescence imaging of invasion outcomes. **a**: Small PV; **b**: Large PV, **c**: Lysis plaque. Bar = 20 μm. *B. besnoiti* tachyzoites are stained with a rabbit anti-Bb antibody and FITC-labelled anti-rabbit antibody resulting in a green fluorescence. Cell nuclei are DAPI stained, resulting in a blue fluorescence

IFAT was carried out on infected MARC-145 monolayers at 72 hpi. The wells were washed 3 times with PBS and cells were fixed with ice-cold methanol for 10 min. After further 3 washes in PBS, specimens were permeabilized by adding 300 μl of PBS containing 0.2 % Triton-X-100 into each well and incubating for 30 min at 37 °C, followed by further 3 washes with PBS. Parasites were then labelled with a rabbit-anti tachyzoite Bb-Spain1 [40] at a dilution of 1:1000 in PBS for 1 h at 37 °C. After further 3 washes with PBS, a secondary antibody conjugate (Alexa Fluor® 488 Goat Anti-Rabbit IgG (H + L) Antibody (Ref. A-11034, Life technologies)) was applied at a dilution of 1:1000 in PBS, and the plates were incubated for 45 min at 37 °C in the dark. They were washed 3 times with PBS, and in the final wash DAPI stain was included. Finally, the plates were washed with distilled water and the invasion rate (IR), i.e. the total number of invasion events per well, was counted using a fluorescence microscope (Nikon eclipse TE200) at 200X magnification. Three categories of invasion outcomes were distinguished (Fig. 1): small parasitophorous vacuoles (PVs), large PVs, and lysis plaques. Vacuoles filled with tachyzoites forming a rosette and with individually distinguishable tachyzoites were regarded as small PVs. When the PV was packed with tachyzoites that were not individually discernible, a large PV was recorded. As soon as the host cell was lysed, manifesting in an accumulation of multiple infected cells with just a few tachyzoites infecting each cell, the infected cells typically being located around a central space with no cells at all and extracellular tachyzoites, a lysis plaque was identified.

### Assessment of proliferation assays by quantitative real-time PCR

Samples in lysis buffer were thawed and incubated for 10 min at 56 °C. DNA was purified using a spin column protocol for cultured cells according to the manufacturer’s instructions (DNeasy kit, Qiagen). DNA was eluted in 50 μl elution buffer. DNA content and purity of each sample was assessed by UV spectrometry (NanoPhotometer®, Implen GmbH, Munich, Germany).

A modified BbRT2 quantitative real-time PCR (qPCR) assay specific for *Besnoitia* spp. DNA from ungulates (i.e., *B. besnoiti, B. tarandi, B. caprae,* and *B. bennetti*; [41]) was performed exactly as described earlier [18]. Primers Bb3 (5’-CAA CAA GAG CAT CGC CTT C-3’; 20 μM) and Bb6 (5’-ATT AAC CAA TCC GTG ATA GCA G-3’; 20 μM) were used. As positive control served DNA extracted from a MARC-145 culture infected with Bb-Spain1, while for the negative control the DNA extraction process was performed on water instead of cells. All samples were run in duplicates in parallel in two PCRs: the BbRT2-PCR, and the 28S-PCR [42] to control for the integrity of the DNA and to detect inhibitory effects. A *Besnoitia*-standard curve corresponding to 10^−1^ to 10^5^ Bb-Spain1 tachyzoites was included in each BbRT2-PCR, which allowed expressing the threshold cycle values (Ct-values) obtained for positive samples as tachyzoites. To account for variations in DNA content of the samples, the number of tachyzoites per μl was normalized using the DNA concentration per μl determined using a NanoPhotometer® (Implen, Munich, Germany), and finally expressed as tachyzoites per nanogram DNA.

### Statistical analyses

To assess differences in time points in invasion and proliferation assays for each isolate, and between isolates, ANOVA tests according to Welch (not assuming equal variances between groups) were performed. In case of significant result comparison between time points, pairwise *t*-test comparison without the use of pooled standard deviation was carried out [43].

To investigate the invasion and proliferation of *Besnoitia* isolates as a function of time, non-linear mixed-effect model methods were employed [44].

For the analysis of invasion rates, we used the total number of invasion events as response variable and the time point of washing as explanatory variable. Invasion kinetics showed an asymptotic curve and was modelled with the following formula:

$$ y=\mathrm{Asym}\left(1-{\mathrm{e}}^{-{\mathrm{e}}^{\mathrm{lrc}}x}\right) $$

Where Asym represents the horizontal asymptote as x tends to infinity and lrc represents the logarithm of the rate constant. The half time, i.e. the time to reach half the value of the asymptote, is then calculated as log 2/*e*^lrc^.

Differences in proportion of invasion outcomes between isolates were assessed by *χ*^2^ statistics, followed by Fisher’s exact test with Bonferroni correction for each pairwise comparison. The relationship between proportions of different invasion outcomes across isolates was assessed by Pearson correlation coefficient.

For the analysis of proliferation rates, we used the number of tachyzoites per nanogram DNA as response variable and the time point of harvesting as explanatory variable. On a first step, data of each isolate were screened to assess general growth pattern. When growth data showed an inflection point and subsequent curve toward a horizontal asymptote we constructed a simple logistic regression model using following formula:

$$ y=\frac{\mathrm{Asym}}{1+{\mathrm{e}}^{\left(\frac{\mathrm{tmid}-x}{\mathrm{scal}}\right)}} $$

Where Asym represents the horizontal asymptote as x tends to infinity, tmid represents the inflection point i.e. the time value at which y is equal to Asym/2, and scal represents the distance between the inflection point and the point for which y is equal to Asym ∕ (1 + e^− 1^), i.e. approximately 0.73 x Asym.

Where growth data showed exponential curve without inflexion point, we constructed an exponential growth model using following formula.

$$ Y={Y}_0\times {\mathrm{e}}^{\mathrm{k}x} $$

Where Y_0_ is the value of Y when x is equal to zero and k is the rate constant. The doubling time (dT), i.e. the time needed so that Y is doubled, was then calculated as ln(2)/k.

To allow comparison between logistic and asymptotic growth curves, we considered the exponential phase of growth in each curve, as in [25].

To account for unexplained variation between experiments, we included the assay as a random factor to the non-linear models. To account for heteroscedasticity, we included a variance structure to each model [44]. Model fit was assessed by inspection of the residuals. Doubling time and half time were calculated by delta method [45]. Statistical analysis was performed with the R statistic software (version 3.0.3; [46]) with the “nlme” package (version 3.1-113, [47]).

## Results

### *B. besnoiti* isolates differ in their invasive capacity

**Fig. 2:**
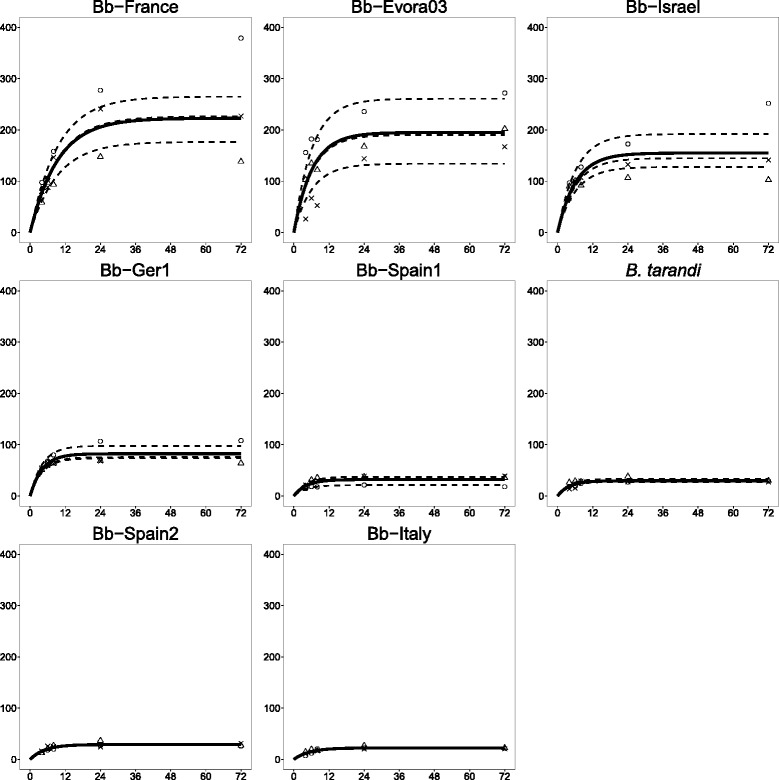
Kinetics of invasion for *B. besnoiti* and *B. tarandi* isolates. Graphs represent number of total invasion events as a function of time in invasion assay. Kinetics of invasion followed an asymptotic curve. Solid lines represent fixed effect. Dashed lines represent assay specific growth curves. Different symbols represent measurements taken during individual replicas. X-axis: hours post infection (hpi); Y-axis: counts of total invasion outcomes per well

Invasion events were homogeneously distributed over the cell monolayer for all isolates (data not shown). Results for Bb-Spain1, which was always included as a control, were comparable in all experimental series, indicating good reproducibility of the experiments (Fig. 2). For all isolates, the invasion kinetics could be modelled as an asymptotic curve (Fig. 2). The maximal IRs significantly differed between the isolates: high invaders (Bb-France, Bb*-*Evora03, and Bb-Israel) achieved IRs of 15–22 % (percentage of inoculated tachyzoites that led to an invasion outcome), while low invaders (Bb-Spain1, Bb-Spain2, Bb-Italy2, and *B. tarandi*) only reached IRs of about 3 %, and studies with Bb-Ger1 revealed an intermediate IR of approx. 8 % (Table 2). Significant differences among isolates were observed at all time points p.i. (Additional file 1: Figure S1).

**Table 2:** Parameters estimated based on asymptotic growth model and influence of time point on total number of events in invasion assay

Isolate	Asym	(CI)	lrc	(CI)	Av velocity	Half time	(CI)
Bb-France	222.8	(154.1–291.6)	−2.2	(−2.6–-1.9)	17.1	6.5	(4.7–8.3)
Bb-Ger1	82.3	(65.6–99.0)	−1.4	(−1.7–-1.1)	14.2	2.9	(2.1–3.6)
Bb-Israel	155.0	(110.8─199.1)	−1.8	(−2.2–-1.4)	18.0	4.3	(2.7–5.8)
Bb-Italy2	22.5	(19.8–25.1)	−1.7	(−2.0–-1.4)	3.1	3.6	(2.6–4.6)
Bb-Evora03	195.0	(122.6–267.7)	−1.8	(−2.2–-1.5)	22.7	4.3	(3.0–5.6)
Bb-Spain1	32.1	(22.0–42.1)	−1.4	(−1.7–-1.2)	5.4	3.0	(2.4–3.5)
Bb-Spain2	29.1	(25.5–32.6)	−1.6	(−1.9–-1.3)	4.3	3.4	(2.5–4.4)
*B. tarandi*	29.7	(24.6–34.9)	−1.4	(−1.7–-1.0)	5.3	2.8	(1.9–3.6)

Asym: asymptote, i.e. maximum of events reached; lrc: logarithm of the rate constant; Av velocity: average velocity of invasion events expressed as invasion events per hour; half time: time after which 50 % of the asymptote was reached

Comparison of IRs at the different time points for each isolate showed that for Bb*-*Spain1 and Bb-Ger1 there were no differences between time points, i.e. after 4 hpi, no statistically significant increase in the number of parasites that had invaded was observed any more. For Bb-Spain2, Bb-Israel, Bb-Evora03, and *B. tarandi*, an increase until 6 hpi was observed, while Bb-France and Bb-Italy2 continued the invasion process until 24 hpi (Fig. 2). Although not statistically significant, an increase in absolute numbers of invaded parasites was observed for all isolates until 24 hpi. This was also reflected by the half time, i.e. the time necessary for 50 % of invasion events to occur, that varied between 2.8 and 6.5 h among the different isolates. The high invaders Bb-France, Bb*-*Evora03, and Bb-Israel exhibited longer half times (4.3–6.5 h), compared to the other isolates (2.8–3.6 h) (Table 2).

**Fig. 3:**
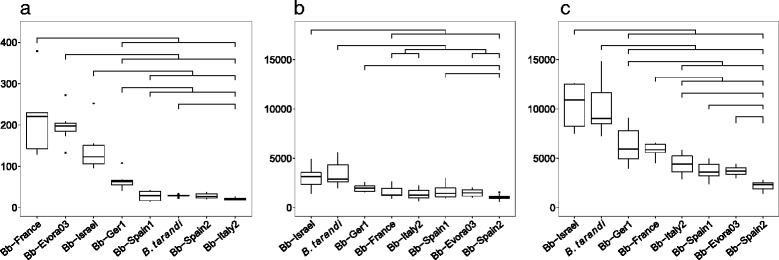
Invasion rates and tachyzoite yield for isolates (box plots). **a**: Total number of total invasion outcomes at 72 hpi. Means and quartiles are presented. Significant differences between the isolates (*p* < 0.05) are indicated. X-axis: isolates; Y-axis: total number of invasion outcomes per well; **b** and **c**: Tachyzoite yields at 72 hpi and 144 hpi in proliferation assay, respectively. Means and quartiles are presented. Significant differences between the isolates (*p* < 0.05) are indicated. X-axis: isolates; Y-axis: estimated number of tachyzoites per ng DNA as determined by qPCR

An average invasion velocity was established for each isolate. The highest velocity was recorded for Bb-Evora03 (22.7 invasion events/h), followed by Bb-Israel, Bb-France, and Bb-Ger1 (14.2–18 events/h) (Table 2). Much lower velocities were recorded for the other isolates Bb-Spain1, Bb-Spain2, Bb-Italy2, and *B. tarandi* (3.1–5.4 events/h) (Table 2). The comparison of IRs of all isolates at 24 hpi is shown in Fig. 3a.

### *B. besnoiti* isolates exhibit distinctly different proliferation kinetics

**Fig. 4:**
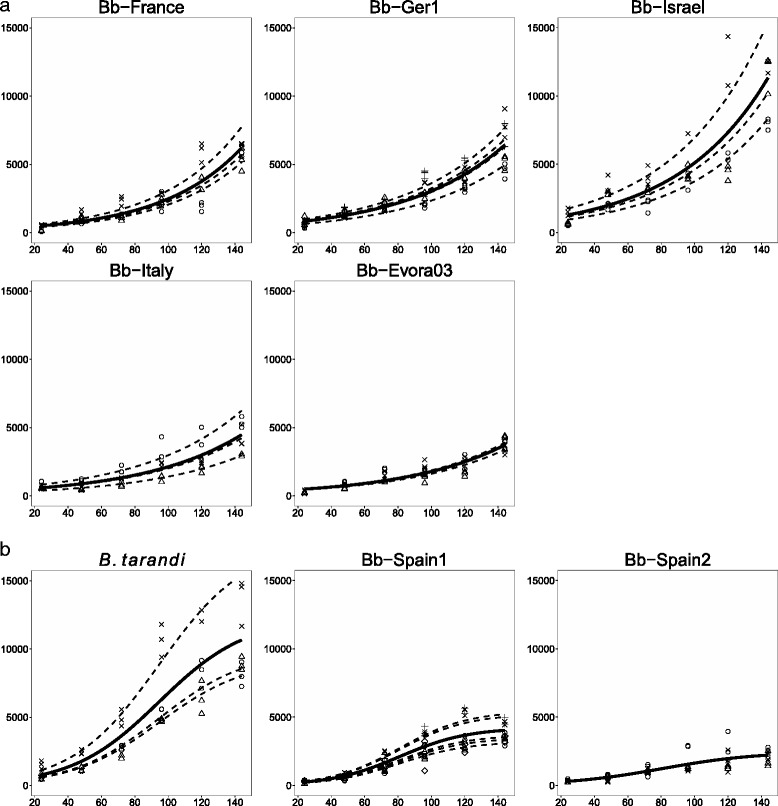
Tachyzoite yield as a function of time for *Besnoitia* isolates showing exponential growth **a** or logistic growth **b** in proliferation assay. Solid lines represent fixed effect. Dashed lines represent assay specific growth. Different symbols represent measurements taken during individual assay replicas. X-axis: hours post infection (hpi); Y-axis: tachyzoites per ng DNA

The proliferation kinetics for each *Besnoitia* isolate was displayed by plotting the parasite loads (as determined by real time PCR) against their respective time points of collection. The growth curves obtained for Bb-Spain1, included as a control in each experimental series, indicated a low variation between the experiments (Fig. 4). Growth curves were fitted to the best matching model. Five isolates exhibited exponential growth, namely Bb-France, Bb-Ger1, Bb-Israel, Bb-Italy2, and Bb-Evora03 (Fig. 4a), and three isolates showed a logistic growth pattern, namely Bb-Spain1, Bb-Spain2, and *B. tarandi* (Fig. 4b, Additional file 2: Table S1). To establish the duration of the lag phase after invasion (i.e. the phase without proliferation after invasion), additional time points 4 hpi and 8 hpi were analysed for Bb-Spain1. As no tachyzoite proliferation was observed for those time points, the growth kinetics for all *Besnoitia* isolates was followed from 24 h onwards. Indeed, all isolates with the exception of Bb-Italy2 and *B. tarandi* exhibited a significant increase in tachyzoite numbers between 24 and 48 hpi (Additional file 3: Figure S2), indicating that their lag phase was not longer than 24 hpi. In *B. tarandi*, the increase was close to significance value (*p* = 0.072), whereas Bb-Italy2 clearly showed a lag phase of 48 h (Additional file 3: Figure S2). Egress of tachyzoites after lysis of their host cells was observed by light microscopy between 48 and 72 hpi for all isolates with the exception of Bb-Italy2, which required 72 to 96 h until parasites egress was noted. The dT was assessed for the exponential growth phase of each isolate, and for one lytic cycle; therefore, time points until 72 hpi were considered for all isolates except for Bb-Italy2, where 48 hpi to 96 hpi time points were considered. The dT was shortest for Bb-Spain 1 (17.9 h), followed by Bb-Evora03 (21.5 h), *B. tarandi* (24.4 h), Bb-France (25.2 h), Bb-Spain2 (26.2 h), Bb-Italy2 (27.1 h), Bb-Ger1 (33.3 h), and Bb-Israel required the longest dT (35.2 h) (Additional file 4: Table S2). The dT of Bb-Israel, Bb-Ger1, Bb-Italy2, Bb-Spain2, and *B. tarandi* was significantly longer than that of Bb-Spain1. In addition, Bb-Ger1 had a significantly longer dT than Bb-Evora03 (Additional file 4: Table S2).

Analysis of the tachyzoite yields after 3 and 6 days of culture showed that Bb-Israel and *B. tarandi* exhibited the highest proliferation rates (Fig. 3b). At day 6 p.i., four groups that statistically differed from each other could be identified (Fig. 3b). Highest values were found for Bb*-*Israel and *B. tarandi* (group 1) (*p* = 0.021), followed by Bb-Ger1 and Bb-France (group 2) and group 3 comprised of Bb-Italy2, Bb-Spain1, and Bb-Evora03. The lowest parasite load was found for Bb-Spain2 (Fig. 3b). Daily microscopical observations every 24 h over 6 days confirmed the values obtained by qPCR: Whereas in Bb-Israel from day 5 on the wells were filled with released tachyzoites, Bb-Spain2 infected cultures still exhibited intact host cell monolayers even after 6 days, alongside lysis plaques with free tachyzoites and intracellular parasites (Additional file 5: Figure S3).

### *B. besnoiti* isolates display different invasion outcomes

**Fig. 5:**
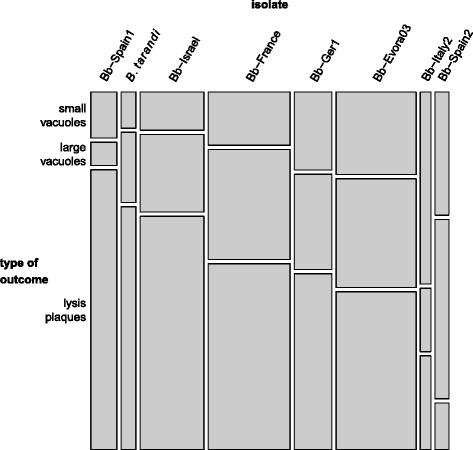
Mosaic plot displaying proportions of invasion outcomes counted per isolate. The area of each tile is proportional to the number of events it represents. Small PVs: four or more tachyzoites forming a rosette with individually distinguishable tachyzoites. Large PVs: tachyzoites were no more individually discernible, but the host cell was still intact. Lysis plaques: accumulation of multiple infected cells with just a few tachyzoites infecting each cell, located around a central space with no cells at all and extracellular tachyzoites

After 72 h, infected cultures exhibited small PVs, large PVs, and lysis plaques. In all isolates, all three possible outcomes were simultaneously present, indicating that *Besnoitia* spp. proliferation is rather asynchronous. Overall, the predominant outcome was the formation of lysis plaques, indicating that one lytic cycle takes about 72 h. Large PVs were less frequently found, and small PVs occurred at even lower numbers. For each isolate the proportions of invasion outcomes observed remained constant, irrespective of the time points assessed (data not shown). The proportions of invasion outcomes significantly differed among isolates (*χ*^2^ = 2758, df = 14; *p*-value < 0.001; Figure 5). In Bb-Spain1, Bb-Israel and *B. tarandi*, 67–81 % of observed outcomes were lysis plaques, and in Bb-France, Bb-Ger1, and Bb-Evora03, lysis plaques accounted for about 50 % of observed outcomes. In Bb-Spain2, the picture was dominated by large PVs that accounted for more than 50 % of all outcomes, followed by small PVs, whereas lysis plaques only made up 13 % of all outcomes. In Bb-Italy2, small PVs were the predominant form (55 % of all outcomes), followed by lysis plaques (28 %) and large PVs (17 %). There was a clear negative correlation between the proportion of lysis plaques and both small PVs and large PVs (Pearson's correlation coefficient: −0.82, and −0.75, respectively). The correlation between the proportions of large PVs and small PVs was slightly positive (Pearson's correlation coefficient: 0.24). However, Bb-Italy2 clearly appeared as an outlier in all three comparisons. This was mainly due to the much higher proportion of small PVs as compared to large PVs in case of this isolate. Exclusion of Bb-Italy2 led to stronger correlations (lysis plaques vs. small PVs: −0.94; lysis plaques vs. large PVs: −0.97; large PVs vs. small PVs: 0.84).

## Discussion

This is the first study that describes the kinetics of the *B. besnoiti* lytic cycle, in order to obtain a standardised in vitro model for the tachyzoite stage. *B. besnoiti* tachyzoites have been successfully propagated in vitro similarly to the closely related *T. gondii* and *N. caninum* (reviewed in [48]). Furthermore, *B. besnoiti* tachyzoite cultures have been used to assess the effects of a limited number of potentially interesting compound classes such as thiazolides and arylimidamines [22, 23] and to determine whether these compounds act against invasion and/or proliferation of *B. besnoiti* tachyzoites. However, the *B. besnoiti* lytic cycle has not been characterised in depth, which is crucial to understand the pathogenesis of this parasitic infection. The currently available data suggest that a large part of the pathogenesis of the acute stage of besnoitiosis is due to repeated host cell invasion and lysis, which leads to the destruction of vascular endothelial cells [49]. These events, combined with a potential direct toxic effect mediated by the parasite, and associated immunopathology, may lead to vascular lesions and increased vascular permeability (reviewed in [21]).

In this study, we have covered seven distinct *B. besnoiti* isolates originating from six different countries, which represent a good part of the isolates available from Europe and Israel.

Until now 15 attempts to grow *B. besnoiti* have been reported (reviewed in [21]). However, only a few studies have assessed the suitability of different mammalian primary cell cultures and permanent cell lines for the isolation and propagation of both *B. besnoiti* and *B. tarandi* species [8, 41, 50–52]. However, data reported in these studies did not allow to identify a preferred cell type. Apart from variations in the protocols employed, parasite growth may also vary in different cell lines depending on the parasite stage (tachyzoites *versus* bradyzoites) inoculated in the cell cultures [41]. Thus, we decided to study the in vitro characteristics of tachyzoites of *Besnoitia* spp. isolates in MARC-145 cells. This cell line was successfully used to isolate *B. besnoiti* from affected cows [39]; this study and allowed long-term cultivation of different *Besnoitia* species [39, 41]; this study. Furthermore, MARC-145 cells were used to assess the in vitro characteristics of different *N. caninum* isolates [25, 36]. Importantly, the invasion efficiencies and growth rates of the different *N. caninum* isolates in MARC-145 cells were positively correlated with the virulence of these isolates in mice [25]. This supports the notion that in vitro cultures represent adequate models to study characteristics of apicomplexan parasites. However, prolonged in vitro culture may alter the features, and most notably the virulence, of isolates, as has been shown for *N. caninum* and for *T. gondii* [53, 54]. Ideally, isolates with limited number of passages after their original isolation should be used for characterisation, and this was the case for Bb-Spain1, Bb-Spain2, Bb-Italy2 and *B. tarandi* in this study. Alternatively, isolates can be passaged through an animal host to overcome the effects of culture adaptation [25]. This approach has been used for Bb-Ger1 and Bb-Evora03, both of which were passaged through interferon-gamma knockout (KO) mice that rapidly succumb to the acute infection [6, 8, 41]. Unfortunately, this was not possible for Bb-Israel and Bb-France. However, the passage number of the Bb-France isolate used in this study was low (20 passages in Vero cells) since this isolate was obtained only in 2013 [55]. In order to overcome this bias of differential adaptation to culture in different cell types, all isolates used here were passaged through the same cell type, namely HFF, prior to the detailed characterization of the lytic cycle in MARC-145 cells.

Regarding the data analysis, the use of mixed-models in parasitological studies is recommended [56]. Such models allow controlling for pseudo-replication where data are grouped, for instance when multiple measurements are taken on the same individual, but also controlling for inevitable variances between repetitions of the same experiment, as applies to this study.

The classical sequential steps of the lytic cycle, such as adhesion-invasion, intracellular proliferation and egress, all of which were previously described for *N. caninum* and *T. gondii*, are also present in *Besnoitia* spp. However, some key parameters of the lytic cycle of *Besnoitia* sp. are clearly distinct from those of *T. gondii* and *N. caninum*. The most striking differences were observed in the IRs of *Besnoitia* sp. as compared with e.g. *N. caninum*: a 5-times higher multiplicity of infection (MOI) in both the proliferation and the invasion assays had to be used for *Besnoitia* compared to similar experiments with *N. caninum* [36]. The values for IRs (5–22 % of tachyzoites led to invasion outcomes) were lower compared to *N. caninum* that had displayed invasion rates of 20–90 % [36] as assessed by the same approach of counting formed vacuoles per added tachyzoites. *Besnoitia* spp. isolates also required more time to invade their host cells compared to *N. caninum* [25, 36]. At 6 h p.i., *B. besnoiti* isolates had completed only 50 % of all invasion events, whereas at this time point *N. caninum* invasion had already reached a plateau (100 %) [25, 36, 57, 58]. A reason for this could be the restricted time of survival in an extracellular environment of *N. caninum* that loses infectivity rapidly within a few hours [58]. For *T. gondii* invasion has been shown to be even more efficient, with 75–80 % of tachyzoites invading their host cells within 30 min p.i. [58]. It should be taken into account that isolates can exhibit a limited invasion and proliferation capacity depending on the host cell type [59]. Indeed, the target cells of *Besnoitia* during natural infection are endothelial cells during the acute phase, and fibroblast and myofibroblasts during the chronic phase of the infection [15, 49]. Accordingly, it would be expected that low invasion rates were correlated with a short extracellular survival period. However, our study clearly showed that *Besnoitia* spp. isolates were capable to actively invade MARC-145 cells during a time-span of up to 24 h, which indicates that extracellular *Besnoitia* tachyzoites can survive and retain their infectivity for extended periods of time. This is in stark contrast to *N. caninum* and *T. gondii*, which invade their host cells within minutes. *N. caninum* tachyzoites loose the capacity to infect their host cells within 2–6 h of extracellular maintenance [25, 57]. Extended extracellular survival of *Besnoitia* spp. tachyzoites might be favourable for mechanical transmission by vectors and in particular tabanids. Experiments investigating survival of *B. besnoiti* in blood sucking insects reported survival of less than 1 h in *Stomoxys calcitrans,* less than 3 h in tsetse flies, and less than 24 h in tabanids (reviewed in [1]).

Following host cell invasion, proliferation of intracellular tachyzoites is initiated. The proliferation assays carried out in this study showed that there is a lag phase of approximately 24 h for Bb-Spain1 (data not shown), and Bb-Italy2 exhibited a lag phase of 48 h. For different *N. caninum* isolates, lag phases of 8–44 h [25, 36], and for the *N. caninum* Nc-1 isolate and *T. gondii* lag phases of 10–12 and 8–10 h have been reported, respectively [60]. Moreover, *B. besnoiti* isolates exhibit a rather extended dT (17.9–35.2 h depending on the isolate). For *N. caninum* isolates, dTs of 9.8–14.1 h [25], and 14–15 h (Nc-1; [60]) were reported. For the *T. gondii* RH strain, an even shorter dT of 8–10 h has been observed [60]. After the lag phase, logistic growth has been described for *T. gondii* and *N. caninum* [60]. Over a period of 144 h, three isolates of *B. besnoiti* also exhibited logistic growth, whereas the other five isolates showed an exponential growth pattern. Exponential growth was also observed by Regidor-Cerillo et al. [25] in different *N. caninum* isolates over a time span of 68 h. However, in this study the observation phase has been 144 h and represented two lytic cycles for most of our isolates; when only the first 72 h are evaluated (e.g. one lytic cycle), all *Besnoitia* isolates also followed an exponential growth pattern.

The significant differences between the isolates in both invasion and proliferation parameters observed in this study suggest that there is a considerable degree of intra-species variability in *B. besnoiti*. Based on the IRs, isolates can be grouped into high invaders (Bb-France, Bb-Evora03 and Bb-Israel), medium invaders (Bb-Ger1) and low invaders (Bb-Spain1, *B. tarandi*, Bb-Spain2 and Bb-Italy2). IRs in *Besnoitia* species appeared to be influenced by invasion velocity as shown by the finding that those invading cells with high velocity also exhibited increased IRs. Interestingly, high IR isolates also showed a prolonged extracellular survival evidenced by higher invasion rates observed at 24 hpi compared to 4 and 6 hpi and, consequently, a higher half time of invasion. Thus, a long invasion period, combined with increased invasion velocity, finally led to highest IRs, whereas those isolates that had a short invasion period, e.g. Bb-Spain1 and *B. tarandi*, did not show a high overall IR.

Another intra-species classification can be carried out when assessing the results of the proliferation assays: according to the tachyzoite yield measured at 72 h p.i., *Besnoitia* isolates can be grouped into three categories: high prolific (Bb-Israel and *B. tarandi*), medium prolific (Bb-Ger1 and Bb-France) and low prolific isolates (Bb-Italy2, Bb-Spain1, Bb-Evora03 and Bb-Spain2). The differences observed among isolates increased after two lytic cycles (at 144 hpi). Isolates displayed two different growth patterns (logistic or exponential) although these were not associated to a prolific category.

Another interesting finding obtained after 72 h of culture of infected cells was the simultaneous presence of small vacuoles, large vacuoles and lysis plaques as evidenced by immunofluorescence. Based on the assumption that small vacuoles transform into large vacuoles and eventually into lysis plaques during the lytic cycle, it is conceivable that those isolates producing predominantly lysis plaques would have a more rapid lytic cycle than isolates displaying predominantly large vacuoles or even small vacuoles at the same time point. This indicates that, in comparison to *T. gondii* and *N. caninum*, the *Besnoitia* spp. lytic cycle is rather more asynchronous. This is likely due to an extended time period during which *Besnoitia* spp. invade their host cells, and the rather extended dTs. After 72 hpi, most isolates had predominantly formed lysis plaques. This was more pronounced for Bb-Spain1, Bb-Israel and *B. tarandi,* the two latter isolates showed high proliferation rates (i.e. tachyzoite yields at 144 hpi). The limited representation of large vacuoles suggests a prompt egression for Bb-Spain1 tachyzoites. In Bb-France, Bb-Ger1, and Bb-Evora03, about 50 % of invaded tachyzoites had already created a lysis plaque; in those isolates, large vacuoles were more often observed than small vacuoles, and these isolates showed lower proliferation rates. In Bb-Spain2, the picture was dominated by large vacuoles, followed by small vacuoles and only few lysis plaques which correlated with the lowest proliferation rate as determined for this isolate. For Bb-Italy2, however, the interpretation of the invasion outcome was less clear; in this isolate, small vacuoles dominated the picture, but there were more lysis plaques than large vacuoles. In part, this may be explained by the lag phase of 48 h. A possible explanation for the invasion pattern of this isolate could be the presence of faster and slower tachyzoites in the inoculum. We might speculate that during repeated in vitro passage of isolates, the tachyzoites will be exposed to a selection pressure that results in the synchronization of the lytic cycle. However, our results suggest otherwise, since we showed that the speed of the lytic cycle seems to become an intrinsic in vitro characteristic of a given isolate. However, we must keep in mind that the results presented here have been obtained in one specific cell line and that different host cells could influence the proliferation kinetics, as e.g. Schares et al. observed different proliferation rates of the same *B. besnoiti* isolate in different cell lines [41].

Interestingly, the tachyzoite yield could not be predicted neither by invasion related parameters (velocity and half time invasion) nor by proliferation parameters (lag phase and dT). In contrast, the IR in *N. caninum* was shown to impact on the in vitro proliferation of *N. caninum* isolates of bovine and canine origin [36].

*B. tarandi* did not differ from the assessed *B. besnoiti* isolates, neither with respect to invasion nor proliferation characteristics. Although one isolate is a very limited sample, the close genetic [6], immunogenic [40], and proteomic [61] relationship between *B. tarandi* and *B. besnoiti* seems to be reflected in comparable in vitro characteristics at least in the model used here.

Differences in in vitro behavior could be attributed to the biological diversity of these *Besnoitia* spp. isolates. However, especially the results obtained with Bb-Israel should be interpreted with caution, since this isolate has been maintained for extended periods in cell culture, which may have led to attenuation of virulence, and adaptation to efficient replication in vitro. For *N. caninum* and *T. gondii* parasites, this effect had been reported earlier [34, 54]. For *N. caninum*, a positive correlation between invasion capacity, tachyzoite yield and in vivo virulence in the mouse model could be shown [25, 36]. If this holds true for *B. besnoiti* as well, Bb-France and Bb-Evora03 would be expected to be the most pathogenic isolates, followed by Bb-Israel and Bb-Ger1. To test this hypothesis, animal experiments would be necessary. Liénard et al. succeeded in provoking clinical besnoitiosis in rabbits upon infection with *B. besnoiti* bradyzoites freshly isolated from a cow [62]. For testing in vivo virulence of isolates kept in culture, however, no standardis ed animal models are yet available. Furthermore, all isolates used in this study had been obtained from clinically affected cows or bulls with noticeable skin lesions, i.e. no indication on differences in virulence could be derived from this. Thus, there is currently no possibility to address virulence in *B. besnoiti* isolates. Genetic heterogeneity of *B. besnoiti* isolates could be another explanation for the different in vitro characteristics observed. In this respect, only very few and somewhat contradictory studies have been published. While Madubata et al. [37] reported genetic heterogeneity between four *B. besnoiti* isolates when performing microsatellite analysis, Gutiérrez-Exposito et al. [38] using the same methods could only find one isolate (Bb-Italy2) that differed from 10 other, homogenous, *B. besnoiti* isolates. This leads to the conclusion that further molecular markers are needed and that those markers need to be applied to a broad range of isolates to characterize *B. besnoiti* population structure.

## Conclusions

In summary, this study provided the basis for studies on the lytic cycle of *Besnoitia* tachyzoites. In vitro characteristics of *Besnoitia* isolates are markedly different from those of the related apicomplexan parasites *N. caninum* and *T. gondii* since lower invasion rates, longer extracellular survival, longer dT and longer lag phase were observed. Moreover, this study was the first comparing in vitro characteristics of different *Besnoitia* isolates showing different invasion efficiency and proliferation rates. Whether in vitro models using primary bovine endothelial cells or fibroblasts i.e. in cells that are natural target cells [15, 41] would reveal the same differences between the isolates observed here will have to be shown by future research. Similarly, further molecular and in vitro studies should aim at comparing all *Besnoitia* species affecting ungulates, i.e. *B. besnoiti, B. tarandi, B. caprae*, and *B. bennetti*, preferably each species being represented by various isolates. We cannot tell at this time to what extent the in vitro characteristics are transferable to in vivo traits as it has been shown for *N. caninum* [25, 36]. Unfortunately, there is no standardised animal model, preferably even a bovine model, available. Therefore, the standardised in vitro model currently is the best available option for studies on isolate-specific characteristics. Development of control tools against bovine besnoitiosis is urgently needed in order to combat more efficiently the spread of the disease. In particular, drugs are promising options to fight against the acute stage of the disease and avoid spread of the parasite by prophylactic treatment of newly purchased animals. In this context, this study represents also a proof-of-concept standardised in vitro model to test drug candidates.

## Additional files

Additional file 1: Figure S1. Comparisons of invasion events of each isolate for each time point (box plots). A: 4 hpi; B: 6 hpi; C: 8 hpi; D: 24 hpi; E: 72 hpi. Means and quartiles are presented. Significant differences between the isolates (*p* < 0.05) are indicated. X-axis: isolates; Y-axis: counts of total invasion events per well. (PDF 40 kb) Additional file 2: Table S1. Parameters estimated based on logistic growth model and influence of time on tachyzoite yield in proliferation assay. (DOCX 15 kb) Additional file 3: Figure S2. Comparisons of tachyzoite yield for each isolate for each time point in proliferation assay (box plots). A: 24 hpi; B: 48 hpi; C: 72 hpi; D: 96 hpi; E: 120 hpi; F: 144 hpi. Means and quartiles are presented. Significant differences between the isolates (*p* < 0.05) are indicated. X-axis: isolates; Y-axis: estimated number of tachyzoites per ng DNA as determined by qPCR. (PDF 41 kb) Additional file 4: Table S2. Parameters estimated based on exponential growth model and comparison of doubling times in proliferation assay up to 72 hpi^$^. (DOCX 14 kb) Additional file 5: Figure S3. Microscopic follow up over 6 days of Marc-145 cell cultures infected with Bb-Spain2 (A), and Bb-Israel (B), respectively. Bar = 50 μm. (PNG 7219 kb)
