# Supplementary figures and images for: Besnoitia besnoiti lytic cycle in vitro and differences in invasion and intracellular proliferation among isolates
Source: Parasit Vectors. 2016 Feb 29;9:115. doi: 10.1186/s13071-016-1405-9 (PMC4772326; doi:10.1186/s13071-016-1405-9)

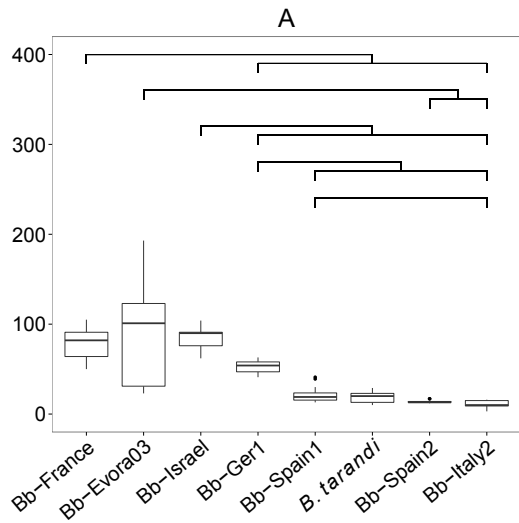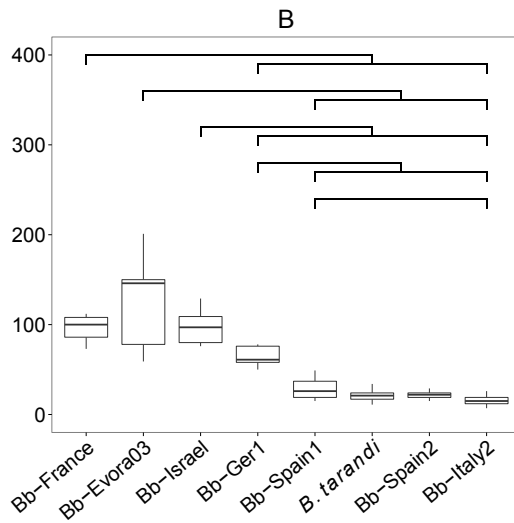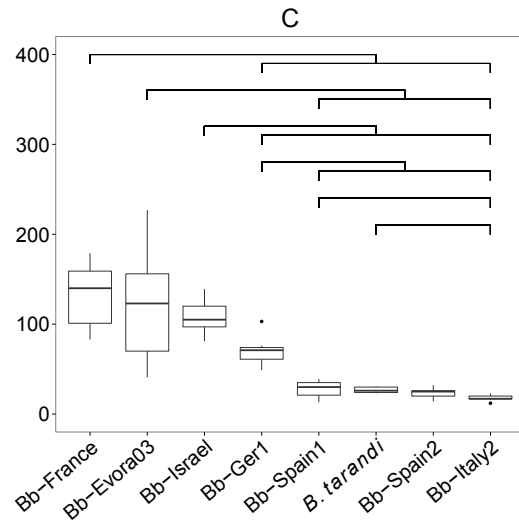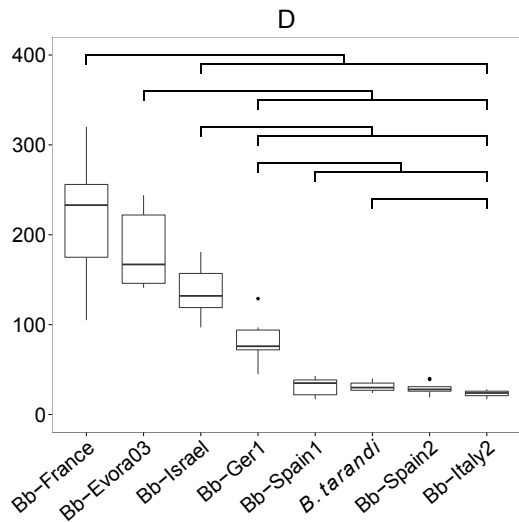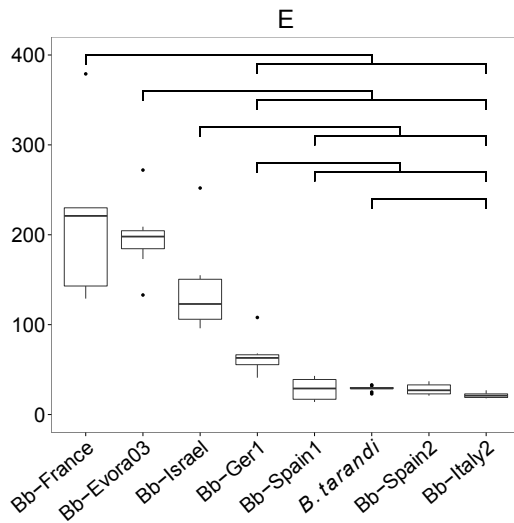

Supplement: Additional file 1: Figure S1. — Comparisons of invasion events of each isolate for each time point (box plots). A: 4 hpi; B: 6 hpi; C: 8 hpi; D: 24 hpi; E: 72 hpi. Means and quartiles are presented. Significant differences between the isolates (p < 0.05) are indicated. X-axis: isolates; Y-axis: counts of total invasion events per well. (PDF 40 kb) [file 13071_2016_1405_MOESM1_ESM.pdf]

A

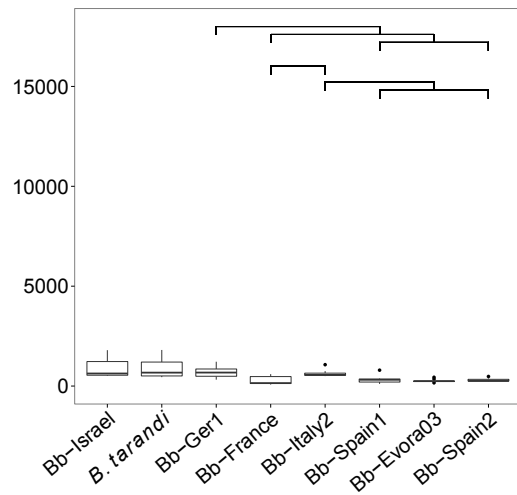

B

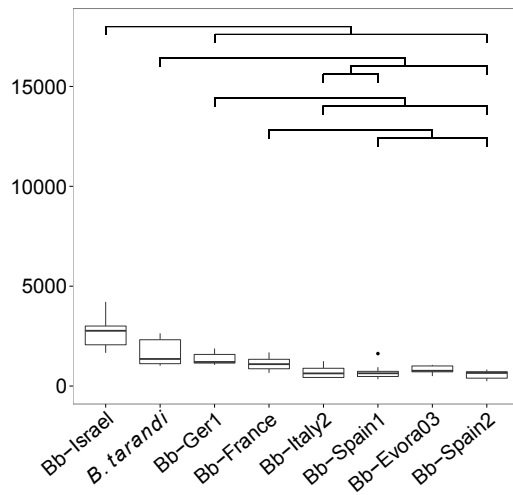

C

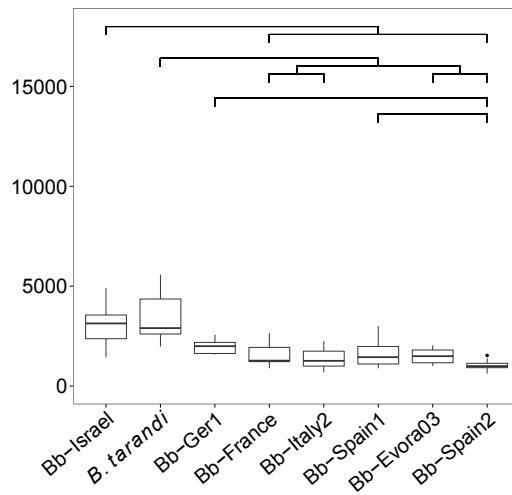

D

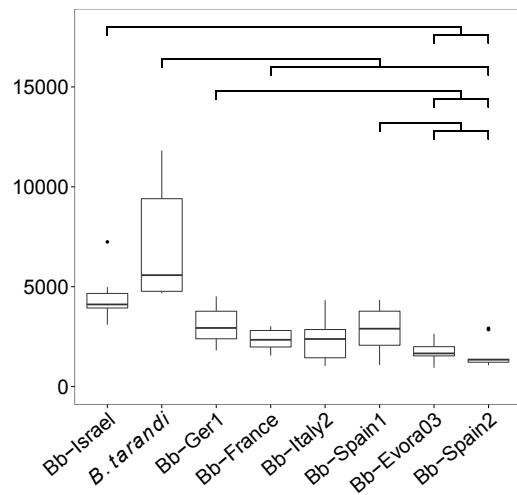

E

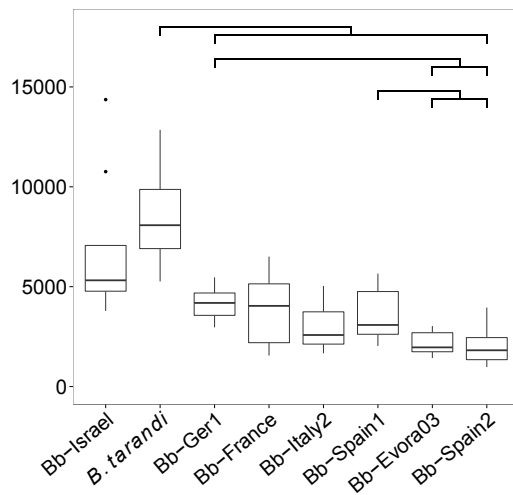

F

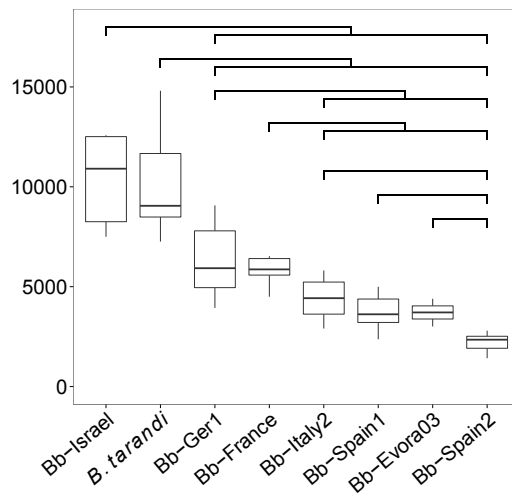

Supplement: Additional file 3: Figure S2. — Comparisons of tachyzoite yield for each isolate for each time point in proliferation assay (box plots). A: 24 hpi; B: 48 hpi; C: 72 hpi; D: 96 hpi; E: 120 hpi; F: 144 hpi. Means and quartiles are presented. Significant differences between the isolates (p < 0.05) are indicated. X-axis: isolates; Y-axis: estimated number of tachyzoites per ng DNA as determined by qPCR. (PDF 41 kb) [file 13071_2016_1405_MOESM3_ESM.pdf]

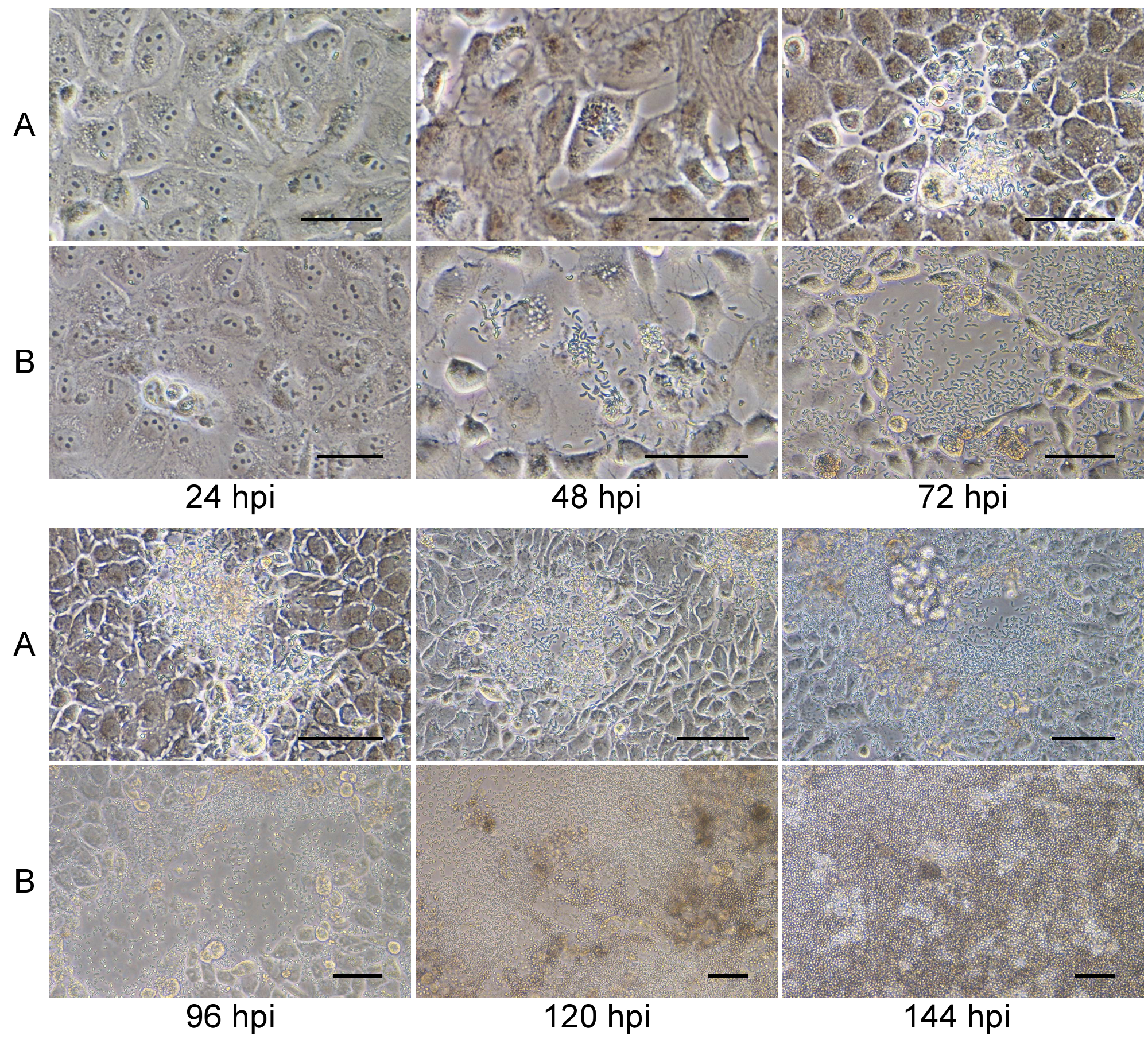

Supplement: Additional file 5: Figure S3. — Microscopic follow up over 6 days of Marc-145 cell cultures infected with Bb-Spain2 (A), and Bb-Israel (B), respectively. Bar = 50 μm. (PNG 7219 kb) [file 13071_2016_1405_MOESM5_ESM.png]
