# Supplementary material for: Besnoitia besnoiti lytic cycle in vitro and differences in invasion and intracellular proliferation among isolates
Source: Parasit Vectors. 2016 Feb 29;9:115. doi: 10.1186/s13071-016-1405-9 (PMC4772326; doi:10.1186/s13071-016-1405-9)
Supplement: Additional file 2: Table S1. — Parameters estimated based on logistic growth model and influence of time on tachyzoite yield in proliferation assay. (DOCX 15 kb) [file 13071_2016_1405_MOESM2_ESM.docx]

**Supplementary Table 1: Parameters estimated based on logistic growth model and influence of time on tachyzoite yield in proliferation assay.**

| **Isolate** |  | **Asym** | **(CI)** |  | **xmid** | **(CI)** |  | **scal** | **(CI)** |
| --- | --- | --- | --- | --- | --- | --- | --- | --- | --- |
| Bb-Spain1 |  | 4251.6 | (3293.6 – 5209.6) |  | 80.6 | (75.5 – 85.7) |  | 20.3 | (18.3 – 22.3) |
| Bb-Spain2 |  | 2367.9 | (1595.7 – 3140.0) |  | 77.2 | (60.1 – 94.3) |  | 27.5 | (21.3 – 33.7) |
| *B. tarandi* |  | 12282.6 | (7454.0 – 17111.3) |  | 94.6 | (85.5 – 103.8) |  | 26.2 | (23.1 – 29.3) |

Asym: horizontal asymptote; xmid: inflection point, i.e. time value at which tachyzoite yield is equal to Asym/2; scal: distance in hours between the inflection point and the point where the response is approximately 0.73 x Asym.
