# Supplementary material for: Besnoitia besnoiti lytic cycle in vitro and differences in invasion and intracellular proliferation among isolates
Source: Parasit Vectors. 2016 Feb 29;9:115. doi: 10.1186/s13071-016-1405-9 (PMC4772326; doi:10.1186/s13071-016-1405-9)
Supplement: Additional file 4: Table S2. — Parameters estimated based on exponential growth model and comparison of doubling times in proliferation assay up to 72 hpi$. (DOCX 14 kb) [file 13071_2016_1405_MOESM4_ESM.docx]

**Supplementary Table 2: Parameters estimated based on exponential growth model and comparison of doubling times in proliferation assay up to 72 hpi^$^.**

| **Isolate** |  | **Y0** | **(CI)** |  | **K** | **(CI)** |  | **Doubling time** | **( CI)** |
| --- | --- | --- | --- | --- | --- | --- | --- | --- | --- |
| Bb-Israel |  | 798.6 | (409.4 – 1187.7) |  | 0.020 | (0.014 – 0.026) |  | 35.2* | (25.3 – 45.0) |
| Bb-Ger1 |  | 457.3 | (340.2 – 574.3) |  | 0.021 | (0.017 – 0.025) |  | 33.3** | (27.3 – 39.4) |
| Bb-Italy2^$^ |  | 204.5 | (104.9 – 304.1) |  | 0.026 | (0.022 – 0.029) |  | 27.1* | (23.9 – 30.3) |
| Bb-Spain2 |  | 159.5 | (109.3 – 209.7) |  | 0.026 | (0.022 – 0.031) |  | 26.3* | (21.8 – 30.8) |
| Bb-France |  | 228.7 | (99.1 – 358.3) |  | 0.027 | (0.021 – 0.034) |  | 25.2 | (19.8 – 30.7) |
| *B. tarandi* |  | 428.0 | (223.4 – 632.5) |  | 0.028 | (0.025 – 0.032) |  | 24.4* | (21.6 – 27.1) |
| Bb-Evora03 |  | 153.8 | (95.7 – 211.8) |  | 0.032 | (0.028 – 0.036) |  | 21.5 | (18.8 – 24.2) |
| Bb-Spain1 |  | 122.1 | (74.7 – 169.4) |  | 0.039 | (0.036 – 0.041) |  | 17.9 | (16.8 – 19.1) |

^$^: for *Bb* Italy, time point 48, 72 and 96 were studied. Y0: calculated number of tachyzoites per ng DNA at time point zero; K: rate constant

* Doubling time significantly different from Bb-Spain1

** Doubling time significantly different from Bb-Spain1 and from Bb-Evora03
